# Celiac Disease Histopathology Recapitulates Hedgehog Downregulation, Consistent with Wound Healing Processes Activation

**DOI:** 10.1371/journal.pone.0144634

**Published:** 2015-12-09

**Authors:** Stefania Senger, Anna Sapone, Maria Rosaria Fiorentino, Giuseppe Mazzarella, Gregory Y. Lauwers, Alessio Fasano

**Affiliations:** 1 Center for Mucosal Immunology and Biology Research, Massachusetts General Hospital for Children and Celiac Program at Harvard Medical School, Charlestown, Massachusetts, United States of America; 2 Celiac Center, Division of Gastroenterology, Department of Medicine, Beth Israel Deaconess Medical Center and Harvard Medical School, Boston, Massachusetts, United States of America; 3 Institute of Food Sciences, National Research Council (CNR), Avellino, 83100, Italy; 4 Department of Pathology, Massachusetts General Hospital, 55 Fruit Street, Boston, Massachusetts, United States of America; National Cancer Institute, UNITED STATES

## Abstract

**Background:**

In celiac disease (CD), intestinal epithelium damage occurs secondary to an immune insult and is characterized by blunting of the villi and crypt hyperplasia. Similarities between Hedgehog (Hh)/BMP4 downregulation, as reported in a mouse model, and CD histopathology, suggest mechanistic involvement of Hh/BMP4/WNT pathways in proliferation and differentiation of immature epithelial cells in the context of human intestinal homeostasis and regeneration after damage. Herein we examined the nature of intestinal crypt hyperplasia and involvement of Hh/BMP4 in CD histopathology.

**Methods and Findings:**

Immunohistochemistry, qPCR and *in situ* hybridization were used to study a cohort of 24 healthy controls (HC) and 24 patients with diagnosed acute celiac disease (A-CD) intestinal biopsies. In A-CD we observed an increase in cells positive for *Leucin-rich repeat-containing G protein-coupled receptor 5* (*LGR5*), an epithelial stem cell specific marker and expansion of WNT responding compartment. Further, we observed alteration in number and distribution of mesenchymal cells, predicted to be part of the intestinal stem cells niche. At the molecular level we found downregulation of indian hedgehog (IHH) and other components of the Hh pathway, but we did not observe a concurrent downregulation of BMP4. However, we observed upregulation of BMPs antagonists, gremlin 1 and gremlin 2.

**Conclusions:**

Our data suggest that acute CD histopathology partially recapitulates the phenotype reported in *Hh* knockdown models. Specifically, Hh/BMP4 paradigm appears to be decoupled in CD, as the expansion of the immature cell population does not occur consequent to downregulation of BMP4. Instead, we provide evidence that upregulation of BMP antagonists play a key role in intestinal crypt hyperplasia. This study sheds light on the molecular mechanisms underlying CD histopathology and the limitations in the use of mouse models for celiac disease.

## Introduction

Intestinal epithelial homeostasis is characterized by continuous crosstalk between epithelium and lamina propria. Studies on adult and developing-intestine [1–3] have identified Hh and BMP signaling pathways as key mediators of this two-way communication. While *Hh* is expressed in the epithelium alone, its target cells reside in the underlying mesenchyme [1]. In the mouse developing-intestine, sonic hedgehog induces the expression of BMP4 [4–6] but not *BMP2* [1]. Knockdown of *Hh* at perinatal age and later, correlates with significant decrease in *BMP4* levels [1, 3] and target-transcription factors *ID1* and *ID3* [3]. BMP deregulation leads to alteration of epithelial proliferation and differentiation including, in the most severe cases, formation of ectopic crypts [2]. These observations suggest that Hh signaling indirectly controls intestinal epithelial stem and immature cell proliferation by modulating BMP4 in the mesenchyme (reviewed in [7]). Furthermore, reduced Hh signaling causes 1) activation of the immune response, including infiltration of neutrophils and macrophages in the lamina propria [3, 8] and 2) remodeling of the predicted niche of the intestinal stem cells (ISC), including reduction of mature smooth muscle cells (SMCs), mislocalization of the intestinal sub-epithelial myofibroblasts (ISEMFs) and expansion of SMC precursor population [9, 10]. Overall Hh downregulation correlates to events consistent with the activation of the wound healing program [3]. Some of the observed phenotypic alterations occurring in *Hh* knockdown mouse strikingly resemble CD [8].

CD is an immune-mediated enteropathy caused by ingestion of gluten in genetically predisposed subjects [11]. The most prominent feature of acute phase CD (A-CD) includes blunting of the villi with loss of mature absorptive cells and crypt hyperplasia, i.e. increased number of mitotically active cells per crypts [12, 13]. The underlying lamina propria shows morphological changes like swelling and infiltration by immune cells such as neutrophils and T lymphocytes.

Gene-expression analyses of intestinal biopsies have shown that key Hh pathway components are downregulated in pediatric A-CD patients [14].

Histological similarities between *Hh* mouse mutants and CD prompted us to further investigate whether other alterations associated with downregulation of Hedgehog/BMP4 pathway occurs in celiac histopathology and to possibly uncover other potentially critical stem cell related pathways that might be altered in acute CD.

## Materials and Methods

### Study design

A total of 48 subjects, 24 healthy controls (HC) and 24 diagnosed acute celiac patients (A-CD) intestinal biopsies, were included in the study. The study was performed retrospectively using biorepository material as part of a protocol to collect extra biopsies for several studies. The applied protocol was approved by local Institutional Review Board (IRB), Massachusetts General Hospital for Children, Boston. The IRB waived the need for patient consent because the data were analyzed anonymously. The study was done in collaboration with the Food Science Institute, CNR, Avellino, Italy. The biopsies obtained from this institution were discharged material obtained for clinically indicated procedures. The protocol was approved by the Ethical Committee of the University Federico II, Napoli, Italy (ethical approval: C.E. n. 230/05). All donors provided written informed consent for the use of the biopsies. The study included standard tissue immunohistochemistry, quantitative gene expression by qPCR and Western blot analysis of protein level.

### Gene expression profile analysis

Total RNA was extracted using TRIZOL reagent #15596018 (Ambion- Life technologies) from duodenal biopsies of 10 HC (5 F, 5 M; mean age: 35.6 ± 7.7) and 10 A-CD patients (6 F, 4 M; mean age: 31.9 ± 7.4). RNA samples (2 μg) were reverse-transcribed to cDNA using random hexamer primers and Maxima universal first strand cDNA synthesis kit #1661 (Thermo Fisher Scientific). Quantitative PCR (qPCR) was performed in an iCycler96X detection system (Bio-Rad, Hercules, CA) according to the manufacturer protocol. RT2 qPCR primers assays for human gene expression profile: *IHH*, *PATCH1*, *HHIP*, *GLI1*, *BMP2*, *BMP4*, *ID1*, *ID3*, *GREM1*, *GREM2* and *18S* genes were purchased from SAB-Qiagen (Qiagen, Limburg, Netherlands).

Difference in gene expression profile was evaluated applying the ΔΔCT method [15], 18S was used as housekeeping gene internal control.

### Protein detection

Western blot analysis was performed as routinely described; briefly, samples were extracted in RIPA buffer, electrophoresed through a 4–20% gradient SDS polyacrylamide gel and transferred onto polyvinylidene difluoride membranes (Millipore, Bedford, MA, USA). Membranes were blocked in blocking buffer (Tris-buffered saline, 0.1% Tween 20, 5% BSA), for 1 h at room temperature. The blots were incubated overnight at 4°C with anti-GREM1 or anti-BMP4 diluted in blocking buffer. After washing, membranes were incubated for 1 h at room temperature with the appropriate secondary antibody diluted in blocking buffer. Western blot signal was visualized using an infrared scan LI-COR Odyssey (LI-COR, Lincon, Nebraska, USA). The signal was quantified using ImageJ software. The protein levels were normalized to beta-actin, internal control.

Immunofluorescence and immunohistochemistry of paraffin embedded duodenal biopsies were performed on well oriented 5μm thick paraffin embedded samples from human duodenum of patients diagnosed with acute celiac disease (A-CD), (N = 6, 3 M, 3 F; median age, 27 years; range 6–70), Marsh grade 3a-c, and normal mucosa subjects (HC), (N = 6, 3M, 3F; median age 31 years; range 3–65), obtained from the Pathology Clinics at MGH. The sections were deparaffinized, rehydrated and treated with 0.3% H_2_O_2_ for 30 minutes to inhibit endogenous peroxidase. Antigen retrieval was performed at high temperature (95°C) in citrate buffer (pH 6.0). Immunostaining was performed according to standard protocol. Images were acquired using an Eclipse 80i microscope (NIKON, Tokyo, Japan).

Antibodies: alpha-smooth muscle actin antibody 1:1000 (#Ab5694, Abcam, Cambridge, UK), desmin antibody 1:50 (#IF02L, Calbiochem-Merck Millipore, San Diego, CA), SOX9 antibody 1:400 (#AB5535 Millipore, San Diego, CA), GREM1 antibody 1:1000 (#ab140010, Abcam, Cambridge, UK), BMP4 antibody 1:500 (#sc-12721, Santa Cruz Biotechnology, Dallas, TX, USA); anti-rabbit Alexa Fluor 488 (#A11034, Life Technologies) and Alexa Fluor 555 anti-mouse (#A21424 Life Technologies) 1:1000; nuclei were counterstained with 6-diamidino-2-phenylindole (DAPI). Vectastain ABC kit (#PK6102, Vector laboratories, Burlingame, CA) was used for DAB detection, according to the manufacturer directions. Biotinylated anti-rabbit IgG #BA-1100 (Vector laboratories, Burlingame, CA) was used in IHC protocol at 1:1000 dilutions. LI-COR IRDye800 anti-rabbit and #92632211, Alexa Fluor 680 goat anti-mouse (#A21057) were used as secondary antibodies in WB analysis (1:5000).

### In situ hybridization

RNAscope Assay kit (Advanced Cell Diagnostic, Hayward, CA) was used to study *LGR5* transcript. Intestinal biopsies from N = 5 HC and N = 5 A-CD samples were used. Hs-*LGR5* probe #311021; Hs-*PPIB* #313901 (positive control) and *DapB* #310043 (negative control), were used. RNAscope 2.0 reagent kit-RED #310036 was used to develop the signal, according to the manufacturer’s instructions.

*In situ* staining signal was scored according to modified manufacturer directions:

0 = No staining or less than 1 dot every 10 cells (40X magnification)
1 = 1–3 dots/cell (20-40X magnification)
2 = 4–10 dots (or fewer dot clusters)/cell (20-40x magnification)
3> 10 dots/cell with distinguishable clusters
4> indistinguishable clusters

### Laser Capture Microscopy and Macroarray analysis

Biopsy specimens were obtained from the distal duodenum of 5 A-CD patients (2M, 3F; median age, 43 years; range 22–77). All A-CD patients were positive for serum anti-endomysial antibodies and from 5 HC (2 M, 3 F; median age, 49 years; range 38–61) with normal intestinal mucosa and negative celiac serology.

Freshly frozen duodenal mucosa samples were cut into 8 μm sections using a cryostat (Leica CM1850; Leica Microsystems, Wetzlar, Germany) and collected on RNase free membrane slides (PEN-membrane, 1 mm glass, Carl Zeiss MicroImaging, Munich, Germany). Immediate fixation in cold acetone was performed prior to staining. Rehydration in water was followed by haematoxylin staining prior to dehydration. The slides were then air dried for 3 min. Microdissection was performed using the Leica LMD 6000 microdissector, to obtain the whole crypts, including pericryptal areas, for each sample. Cells derived from laser capture microdissection were immediately blocked at 42°C for 30’, centrifuged at 3000 rpm for 2’ and stored at -80°C.

The samples were analyzed by quantitative PCR array, according to the manufacturer protocol (Stem cell signaling PCR Array PAHS-047Z, SABiosciences-Qiagen). Software provided by the manufacturer was used to evaluate: amplification quality, genomic contamination, fold change and to generate the Volcano plot. The gene expression was normalized to an internal control equal to the average of 5 housekeeping genes: *ACTB*, *B2M*, *GAPDH*, *HPRT1 RPLP0*.

### Statistical analyses

All statistical analyses used Student *t*-test to evaluate statistical significance. Values of *P <* .*05* were considered significant. Data are displayed as mean ± SD.

## Results

### Hh signaling, but not BMP4, is downregulated in adult acute CD patients

**Fig 1:**
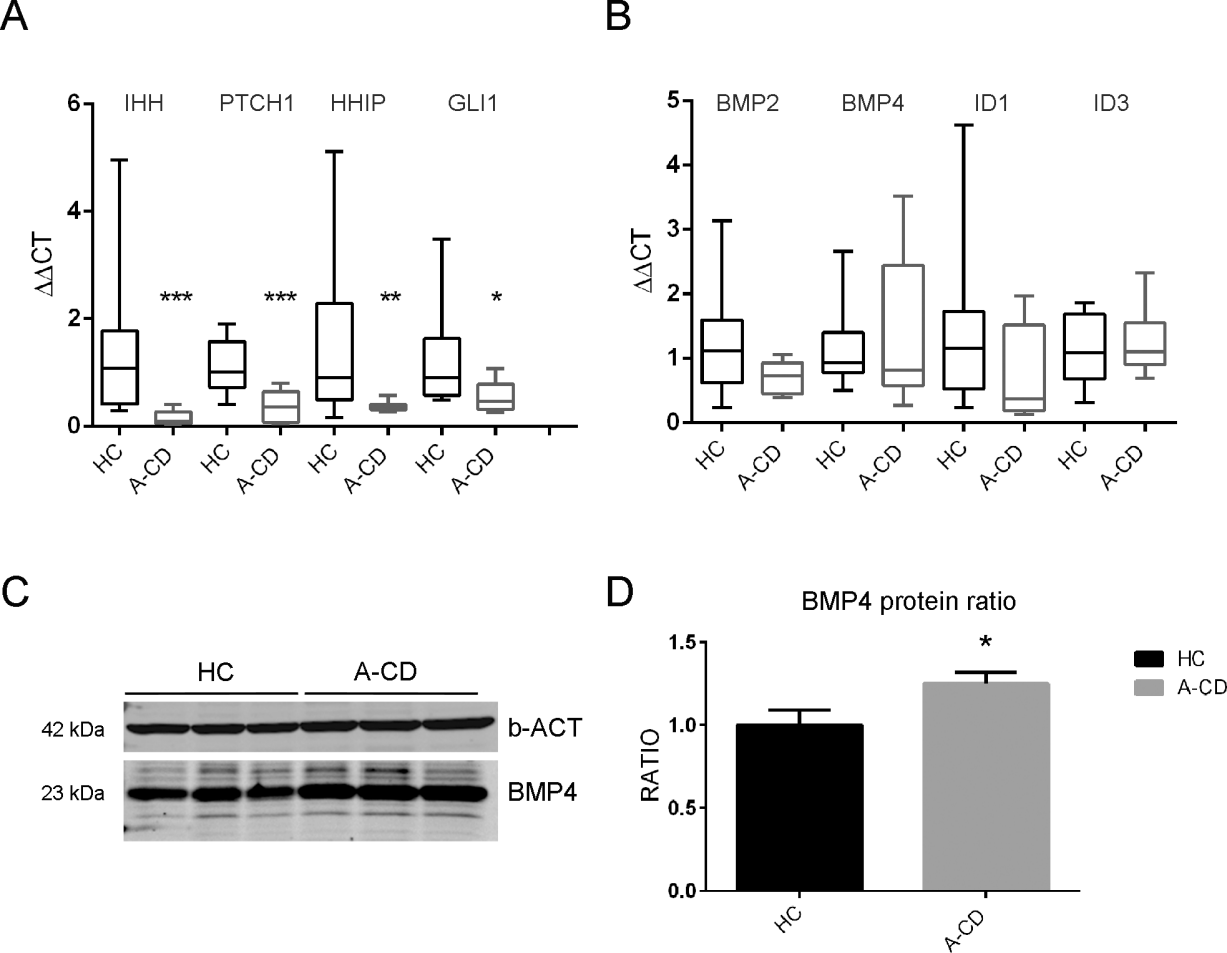
Hh and BMP pathways in intestinal biopsies. Quantitative expression of (*A*) Hh and (*B*) BMP pathway genes in whole duodenal biopsies of HC (N = 10) and A-CD (N = 10). Data are represented as ΔΔCT normalized to the HC mean. (*C*) Western blot of BMP4 protein in total protein extracts of duodenal biopsies in HC (N = 3) and A-CD (N = 3). (*D*) Densitometric quantification of BMP4 protein levels normalized to beta-actin loading control. Data are represented as a mean ± SD. (*) *P <* .*05*, (**) P < .*01*, (***) P < .*001*.

Hedgehog family consists of three members that are differently expressed in the intestinal tract. IHH has been shown to be the most abundant in the adult small intestine (reviewed in [16]). To evaluate the alteration of Hh pathway in CD, we measured the expression of indian hedgehog (*IHH*) and functionally related genes in whole duodenal biopsies from HC, and A-CD by qRT-PCR. *IHH* was found to be significantly downregulated (Fig 1A). Consistent with this result, the expression of IHH target-genes, *PTCH1* (receptor), *HHIP* (Hh interacting protein) and *GLI1* (transcription factor) was significantly decreased in CD subjects compared to controls (Fig 1A). Since lower Hh levels predict lower BMP4 expression as seen previously in mice, the expression of the BMP pathway genes was also analyzed in A-CD samples. Interestingly, no change in *BMP4* expression was detected (Fig 1B), a result that was in contrast with similar analysis previously reported in pediatric A-CD patients [14] and in mouse *Hh* knockdown in which *BMP4* was shown to be the most important target and effector of Hh signal transduction cascade [3]. *BMP2* and *ID1* were downregulated, but not significantly, whereas *ID3* was not affected (Fig 1B). Western blot analysis in whole biopsies revealed a mild but significant increase of BMP4 protein in celiac biopsies (Fig 1C and 1D).

### Lamina propria stromal cells undergo rearrangement following acute insult in CD

SMCs and ISEMFs are directly modulated by Hh in both adult and fetal murine intestine [1, 10]. Chronic reduction of Hh in a transgenic mouse model causes reduction of mature SMCs in the villus core and muscularis mucosa together with expansion of SMC precursors, mislocalization and reduction of ISEMFs [1, 9, 10]. Previous studies in acute celiac small intestinal biopsies reported misarranged and occasionally reduced number of SMCs in the villus area [17].

**Fig 2:**
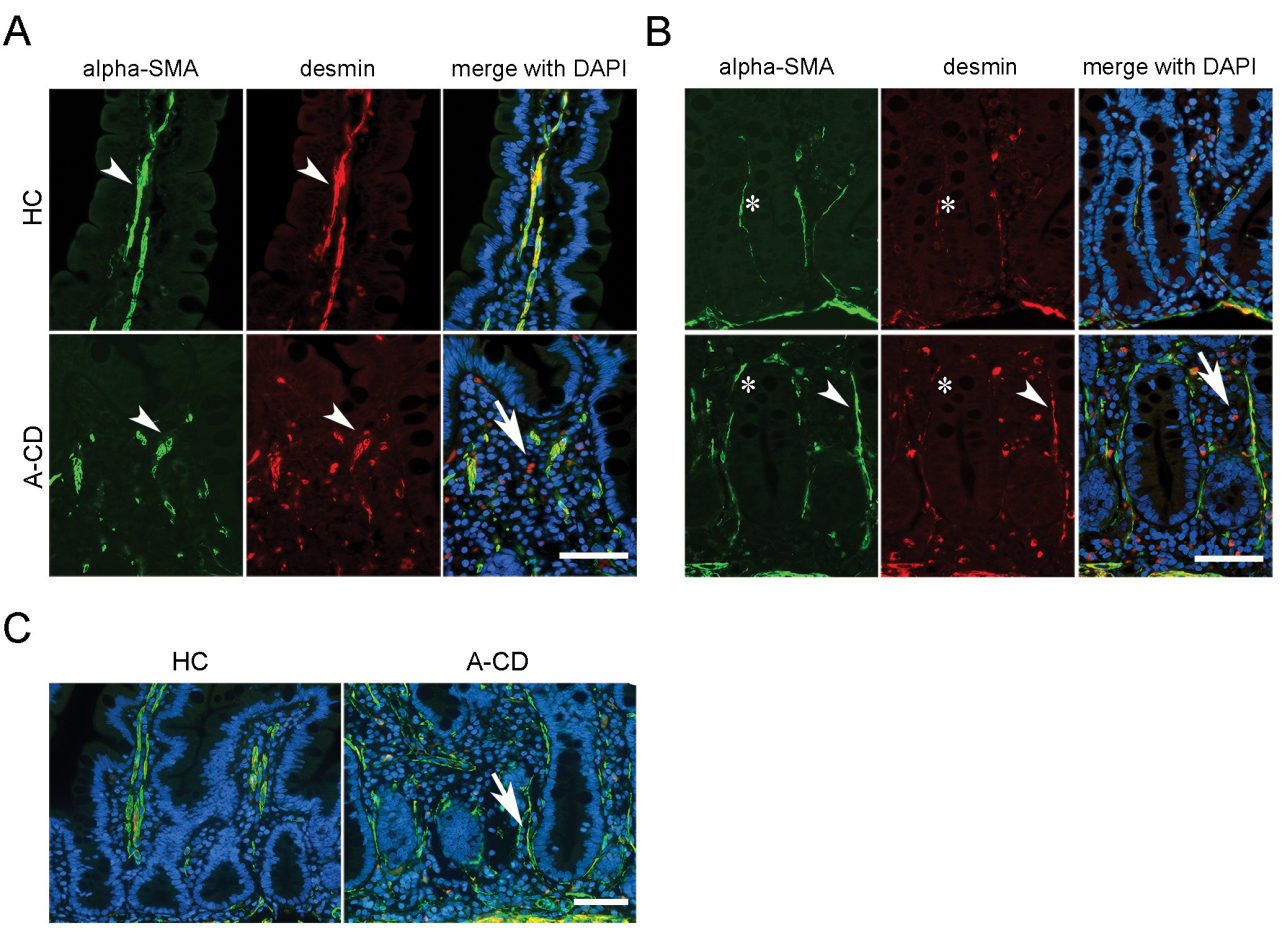
Lamina propria architecture in A-CD patients. Small intestinal biopsies were immunostained with alpha-SMA (green), desmin (red) and DAPI (blue) to visualize SMC, SMC precursor, ISEMF and nuclei. (*A*) Arrowheads indicate SMC fibers positive for alpha-SMA and desmin in HC (top) and A-CD (bottom) villus core. Arrow indicates desmin-only positive round-shaped cell representative of SMC precursors. (B) Asterisks indicate ISEMFs in the crypt region of HC (top) and A-CD (bottom). Arrowheads indicate SMCs along the crypt in A-CD. Arrow indicates desmin-only positive round-shaped cell representative of SMC precursors. (*C*) Lower magnification of exemplary intestinal biopsy of HC and A-CD. Normal villus architecture is visible in HC with well defined SMC fibers along the villus axis. In A-CD, swelling of the mucosae is accompanied by crypt hypertrophy and loss of villus structure. ISEMFs are indicated by arrow. *Scale bar* = 50μm.

Given the importance of mesenchymal cells in contributing to the intestinal stem cells niche, we investigated the distribution and frequency of the lamina propria stromal cells during the acute phase of CD. Normally, mature SMCs are organized in fibers along the villus core and muscularis mucosa. They express *ACTA2* gene (alpha-SMA) that encodes alpha-actin-2 and *DES* gene (desmin) that encodes a muscle specific type III intermediate filament. SMC precursors, however, are rounded in shape, desmin (+) and alpha-SMA (-). ISEMFs, in healthy subjects, are elongated cells organized in a 2–3 cells thick layer around the crypt and in a single cell layer only in the villus. ISEMFs surrounding the crypt robustly express *alpha-SMA* and are very weakly positive or negative for desmin. In the villus area, ISEMFs loose the alpha-SMA positivity and retain ony a weak desmin positive staining (reviewed in [18]). Properly oriented biopsies of HC (N = 6) and A-CD patients (N = 6) were immunostained with anti alpha-SMA, desmin and DAPI (Fig 2). We observed reduced elongated smooth muscle fibers in the villus core of A-CD biopsies compared to HC (Fig 2A) and increased number of rounded desmin (+), alpha-SMA (-) cells, consistent with augmented SMC precursors in both the villus and crypt area (Fig 2A and 2B). We also observed a direct correlation between the extents of tissue damage [11] and aberrant SMCs in the villus. However, we did not observe reduced SMC fibers in the muscularis mucosa (data not shown), in contrast with data in *Hh* knockdown mouse [9].

Finally, we observed a shift of ISEMFs toward the top of the crypt (Fig 2B and 2C) and an overall increase in alpha-SMA positive cells throughout the lamina propria in A-CD (Fig 2C).

### BMP antagonists *GREM1* and *GREM2* are upregulated in acute CD

**Fig 3:**
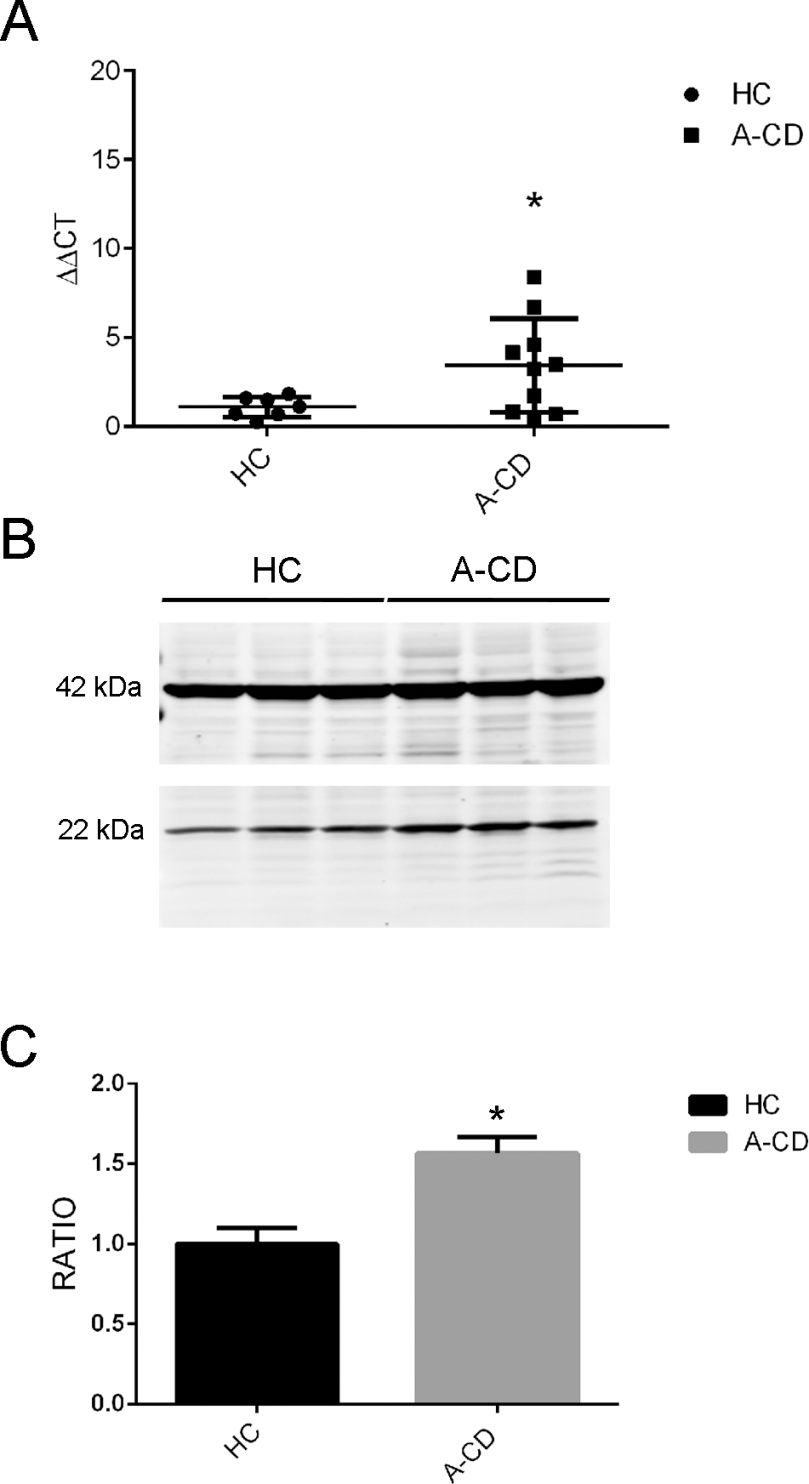
BMP antagonist GREM1 in A-CD. **(**A) *GREM1* expression in whole duodenal biopsies in HC (N = 10) and A-CD (N = 10). Data are represented as ΔΔCT normalized to the HC mean. (B) Western blot of GREM1 protein in total protein extracts of duodenal biopsies in HC (N = 3) and A-CD (N = 3) quantified by densitometry (C) compared to beta-actin protein level as internal control. Data are represented as a mean ± SD. (*) *P <* .*05*.

We further investigated the hypothesis that expansion of the ISEMF domain concurrent with reduced Hh signaling might contribute to crypt hyperplasia in A-CD. ISEMFs are thought to be part of the intestinal stem cell niche providing a proliferative environment to the stem and immature progenitor cells [19]. The current paradigm based on data from *Hh* knockdown mice supports the notion that expanded intestinal crypts is an indirect effect of Hh pathway downregulation that causes a reduction of BMP4 signaling. Under physiological conditions BMP4 limits the intestinal stem cell compartment by antagonizing WNT signaling required for stem and progenitor cell proliferation. Smooth muscle cells and ISEMFs provide a stem cell niche secreting BMP antagonists thus neutralizing BMPs [19] (S1 Fig). Because of the observed lack of BMP4 reduction in A-CD, we tested the hypothesis that BMP antagonists may be upregulated in A-CD. We evaluated two BMP antagonists, GREM1 and GREM2 that are expressed in the intestinal crypt [19]. qRT-PCR and western blot analyses showed that both *GREM1* transcript (Fig 3A) as well as GREM1 protein (Fig 3B and 3C) were significantly upregulated in A-CD intestinal biopsies. *GREM 2* expression was also significantly upregulated (S2 Fig).

### *LGR5* ^*+*^ and WNT responding cells are increased in acute CD

**Fig 4:**
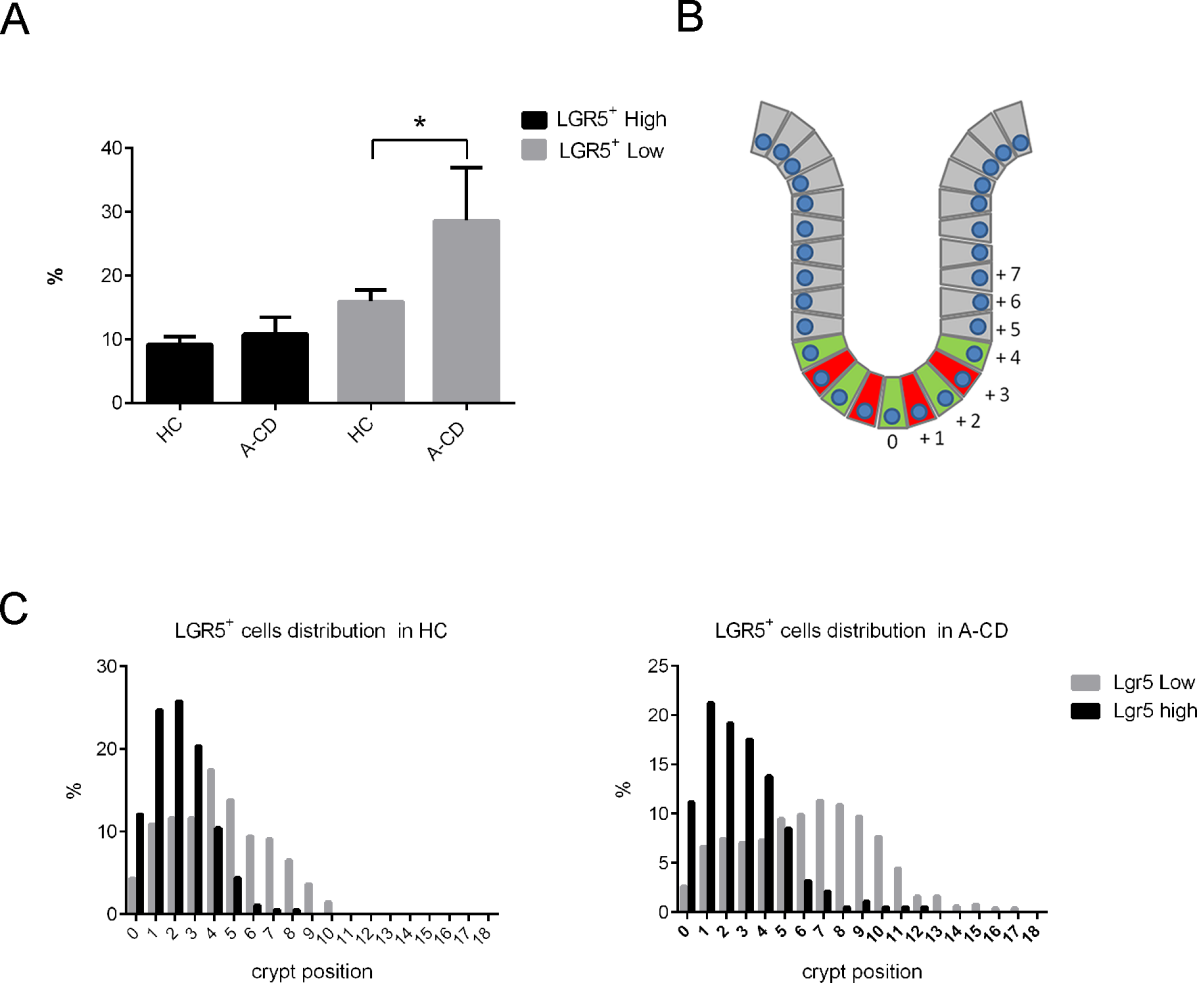
Intestinal epithelial stem cell compartment in A-CD. *In situ* hybridization of *LGR5* transcripts in intestinal biopsies of HC (N = 5) and A-CD (N = 5). *LGR5* ^*+*^ *high* cells and *LGR5* ^*+*^ *low* cells were evaluated based on the criteria described in Material and Methods. An average of 10 crypts per sample was scored. (A) Percentages of *LGR5* ^*+*^ *high* cells (scored 3 and 4) and *LGR* ^*+*^ *5 low* (scored 1 and 2) in HC and A-CD. (B) Schema of an intestinal crypt. Position 0 was arbitrarily assigned to the cell at the bottom of the crypt, where Paneth cells are easily identified; progressive numbers were assigned to adjacent positions moving toward the crypt top. Stem cells are represented in green intermingled with Paneth cells in red. *LGR5* ^+^cells were scored based on their position in the crypt. (C) The distribution of *LGR5* ^*+*^ *low* (gray) and *high* (black) in intestinal crypts of HC and A-CD. Data are represented as a mean ± SD. (*) *P <* .*05*.

Expansion of stem and immature progenitor cells, observed in most severe *Ihh* mouse knockout models [9], prompted us to further investigate the nature of the hyperplasic crypt in CD biopsies. We took advantage of the specificity of *LGR5* intestinal stem cell marker [20]. *LGR5* is highly expressed by stem cells known as crypt-base columnar (CBC) cells that reside at the bottom of the intestinal crypt (Fig 4 *B*). CBCs continuously proliferate and give rise to progenitor cells (Transit Amplifying, TA cells), that express low levels of LGR5 and occupy the remaining part of the crypt. TA cells differentiate into all the mature epithelial cells types (reviewed in [21]). *In situ* hybridization was performed on properly oriented biopsy sections to identify and quantify the stem cells (*LGR5high*) and immature TA progenitor (*LGR5low*) in both HC and A-CD patients. Only crypts with clearly visible Paneth cells were scored for *LGR5* positive cells (S3 Fig).

No significant difference in *LGR5High* cells was detected between the two analyzed groups, suggesting no increase in intestinal stem cell population in CD patients. Conversely we found an increased percentage of *LGR5Low* cells in A-CD, consistent with augmented immature TA progenitor cells (Fig 4A).

**Fig 5:**
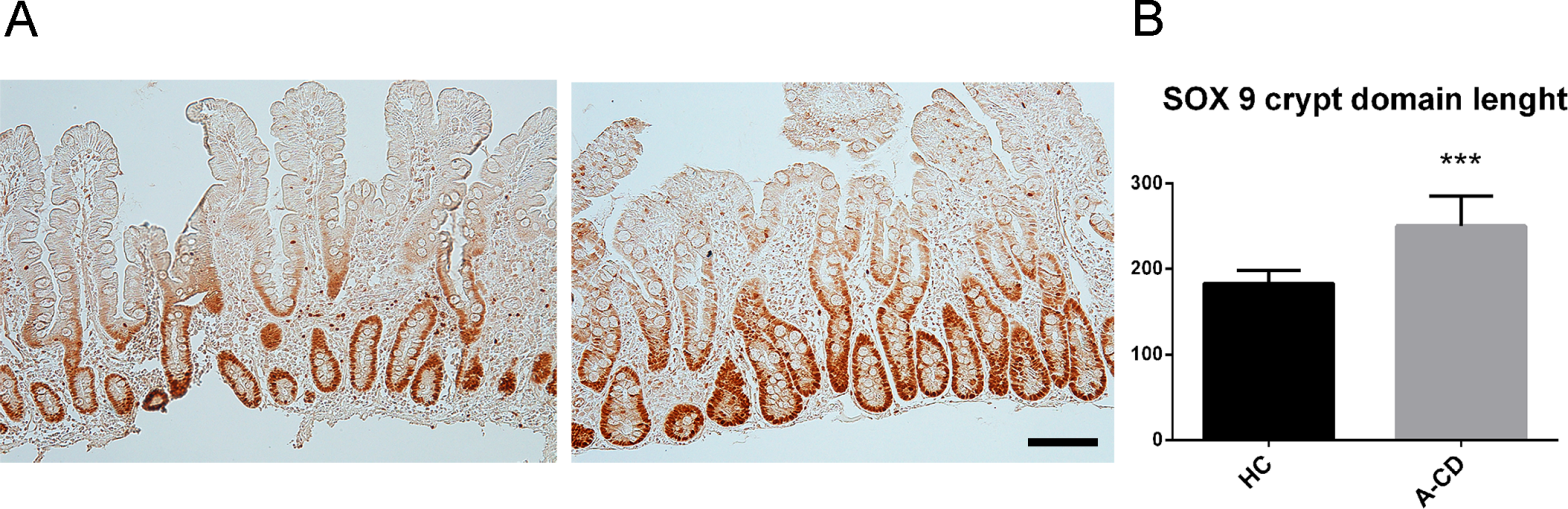
SOX9 in HC and A-CD. (A) IHC of SOX9 was performed on (N = 6) HC and (N = 6) A-CD to evaluate the WNT responding cell compartment. (B) The length of the SOX9^**+**^ cell domain was measured in N = 3 samples per group using NIS-Elements software and is represented in μm. An average of eight crypts per sample was scored. *Scale bar* = 100μm. Data are represented as a mean ± SD. (***) *P <* .*001*.

We further investigated the localization of the stem cells inside the crypts (Fig 4C). The distribution of *LGR5* positive cells was evaluated in HC and compared to similar data obtained for mouse intestinal crypt [20, 22]. As observed in mice, most of the *LGR5High* cells reside at the bottom of the human intestinal crypt, intermingled with Paneth cells, up to position +4, with few outliers (Fig 4C). Similarly, in A-CD, the majority of *LGR5High* cells were found at the bottom of the crypt (Fig 4C). However *LGR5High* cells were found above position +4 with higher frequency in A-CD, suggesting spreading of the stem cell compartment toward the upper crypt. *LGR5low* cells were found up to position 10 in HC, while they were detected up to position 17 in A-CD (Fig 4C). LGR5 is a transmembrane co-receptor of WNT and contributes to the maintenance and proliferation of stem/immature intestinal epithelial cells [20]. Increased number of *LGR5 positive* cells is consistent with WNT signaling expansion consequent to Hh/BMP signaling reduction [9]. In order to investigate whether WNT compartment is indeed expanded in CD, we performed immunohistochemistry to detect WNT controlled SOX9 transcription factor [23]. As anticipated we found a significantly expanded SOX9 (Fig 5A and 5B) cell compartment in A-CD, consistent with *LGR5* expression data.

### Intestinal stem cell genes altered in celiac intestinal crypts

A-CD offers the opportunity to study the array of genes dysregulated during the acute damage and repair process of the intestinal epithelial lining. Although most of the work done in the mouse model has shed light on basic pathways involved in intestinal stem cell homeostasis, as a result of knockdown of a single gene, it is still unclear whether this information can be extrapolated to the pathology of CD, where multiple factors may contribute to intestinal stem cell regulation.

In order to capture the transcription profile associated with the crypt, rather than bulk tissue, intestinal epithelial and stromal component of the crypt from HC and A-CD were collected together by laser capture dissection microscopy. These samples were analyzed by qPCR for the expression of a stem cell panel of 80 genes. The top 17 differentially expressed genes are summarized in Table 1. Four genes *GLI1*, *GLI3*, *SMO*, *PTCH1* belonging to Hh pathway were significantly downregulated, confirming that Hh pathway is indeed downregulated in A-CD at the crypt level as well. Two components of the Notch pathway, *NCSTN* and *NOTCH4* were also downregulated, however only *NOTCH4* reached statistical significance. *NFAT5* and *LIFR*, negative regulators of the WNT signaling pathway and *BCL9* and *FZD5*, both coupled to the beta-catenin canonical signaling pathway, were also significantly downregulated. Finally, we found seven genes of the TGF-beta/BMP/Activin gene superfamily to be significantly altered in celiac crypts. These genes included the TGF-beta/Activin receptors and co-receptors *ENG* and *TGFBR3*, cytoplasm mediators *SMAD3*,*4*,*9* and the DNA-binding transcriptional modulators, *ZEB2* and *EP300* (Table 1 and S4 Fig).

**Table 1:** Stem cell related gene list altered in A-CD crypts.

***GENE***	**Fold Regulation**	**P-Value**
*BCL9*	-3.8523	0.042667
*ENG*	-2.9705	0.045796
*EP300*	-2.8446	0.010392
*FZD5*	-3.4419	0.034942
*GLI1*	-3.8191	0.019727
*GLI3*	-2.7957	0.025196
*LIFR*	-4.201	0.002577
*NFAT5*	-2.6085	0.007741
*NOTCH4*	-3.8858	0.029935
*PTCH1*	-2.8943	0.032864
*SMAD3*	-2.5416	0.039593
*SMAD4*	-2.5196	0.015226
*SMAD9*	-3.2394	0.011844
*SMO*	-2.6312	0.045707
*TCF7L1*	-4.0579	0.011192
*TGFBR3*	-2.7477	0.011667
*ZEB2*	-3.9196	0.044099

Quantitative gene expression analysis of genes associated with stem cell function, was performed on laser captured crypt samples from HC (N = 5) and A-CD (N = 5). Statistically significant altered genes in A-CD crypts compared to HC are listed. Gene fold regulation and *P-value* is reported for each gene.

## Discussion

Crypt hyperplasia, a hallmark of CD, has been hypothesized to be the consequence of an imbalance between continuous tissue damage due to the mucosal autoimmune insult and inability of the stem cells to compensate [24]. Studies in animal models have demonstrated the requirement of the Hh pathway in intestinal epithelium homeostasis including modulation of stem cell function [7]. With this work, we suggest a mechanistic explanation for hyperplasic crypts in A-CD, and uncover previously unrecognized molecular and histological features of A-CD enteropathy when compared with an *Hh* knockdown mouse. We show that the celiac hyperplasic crypt is indeed characterized by an expansion of the immature progenitor cells compartment as defined by the *LGR5* stem cell marker [25, 26], but no significant expansion of the intestinal stem cell population [25], that was occasionally found misplaced toward the upper side of the crypt. Furthermore, all cells populating the hyperplasic crypt in A-CD were SOX9 positive, suggesting an expanded WNT-responding compartment [23], as seen in the *Hh* knockdown mice [9].

Analyses of gene expression profiles provide evidence of Hh signaling cascade downregulation. However BMP4, the most important effector of Hh pathway, was found unaffected, in contrast to similar studies in both CD pediatric patients [14], and *Hh* knockdown mouse [1, 3], where BMP4 was found significantly downregulated.

Whether the discrepancy between our data and those previously reported in CD [14] is due to physiological differences in patient cohorts or to technical variability needs further investigation. Nonetheless, in line with data from *Hh* knockdown mouse [9], we observe a significant upregulation of the BMP4 antagonist GREM1. We also uncover significant upregulation of the paralog GREM2.

*GREM1* and *GREM2* are expressed in human ISEMFs and SMC and contribute to maintenance of a BMP-free proliferative milieu in the intestinal stem cell niche [9, 19]. Previous studies in mouse limb bud development have shown that the cooperation between Hh and BMP4 signaling is required in *Grem1* gene expression and that GLI3 acts as a *Grem1* repressor [27]. In line with those studies, we report here that three genes belonging to Hh pathway, *GLI1*, *GLI3*, and *SMO* were significantly downregulated at the crypt level in A-CD, suggesting that Hh signaling and specifically GLI3 may also be critical for *GREM1* modulation in the intestinal crypt.

The histological alterations of the villus SMCs and mislocalization of ISEMFs are consistent with observations reported in Hh knockdown mutant. However in A-CD, ISEMFs are much more abundant and scattered throughout the intestinal biopsy, similar to what is observed in chronic or recurrent inflammation [28, 29]. In contrast to the Hh knockdown model [3, 8], CD intestinal mucosa is characterized by higher intraepithelial lymphocytes, infiltration of other adaptive immune cells [30, 31] and high levels of the pro-inflammatory cytokine INF-gamma [32, 33]. Importantly, INF-gamma has been shown to modulate the expression of alpha-SMA in SMCs and myofibroblasts [34–36]. Based on our observations as well as those of others, we speculate that the pro-inflammatory milieu together with Hh downregulation, contribute to the alteration of the stromal cells observed in A-CD.

Finally, the gene expression analysis of stem cell regulating genes performed on crypt samples reveals that both the Hh and NOTCH pathways were altered in A-CD crypt, consistent with published data in CD patients [14, 37]. Several genes belonging to the TGF-beta/Activin/BMP superfamily were also affected. Among them only SMAD3 was previously reported to be downregulated in *Hh* knockdown mouse intestinal crypts [3]. The expression of genes that are predicted to be involved in WNT regulation, were also found significantly altered. Whether these alterations are only transient and characteristic of the acute phase or representing specific genetic features of CD patients is an exciting open question that remains to be addressed. Taken together our data suggest that in A-CD, crypt hyperplasia is driven by expansion of WNT responding compartment consequent to downregulation of global Hh pathway and upregulation of BMP antagonists *GREM1* and *GREM2*, rather than by downregulation of *BMP4* (S1 Fig). Furthermore, our data imply a functional decoupling of Hh and BMP4 pathways in the maintenance of human intestinal gut homeostasis, in contrast to the mouse models. Nevertheless, *BMP2*, that is structurally related to *BMP4*, and the transcription factor *ID1* were, although not significantly, less expressed in CD. Based on our results it is conceivable that BMP2/ID1 may play a role in maintaining Hh signaling in human compared to mouse intestine.

In conclusion, our data describe novel histopathologic and molecular features of CD enteropathy. Validation on larger patient populations is needed for possible diagnostic and therapeutic applicability in patient stratification for the treatment of those patients with persistent intestinal damage despite compliance to a gluten free diet [38, 39]. Specifically, although ~70% of patients with acute CD recover a normal intestinal histology after they are on gluten free diet, there is still a significant number that recover symptomatically, but retain intestinal damage. These individuals are at higher risk of developing severe complications and/or co-morbidities, including intestinal lymphoma [40]. Finally this study provides insight to other enteropathies with histopathology similar to CD including environmental [41] and autoimmune [42] enteropathy in which we can hypothesize alteration of epithelial and underlining mesenchyme cross-communication.

## Supporting Information

S1 Fig Model of intestinal crypt signaling between intestinal stem cell and stem cell niche. (A) At the homeostasis IHH is released by the mature epithelium and received by stromal cells that under IHH stimulus secrete BMP4. It is hypothesized that BMP4 gradient originating from stromal cells along the crypt villus axis opposes to WNT to restrain intestinal stem cell proliferation. WNT is released by Paneth cells and specialized ISEMFs (purple) that secrete also BMPs antagonists GREM1 and GREM2 providing a BMPs free stem cell niche. In *Hh* knockdown mouse a dampening of Hh signaling and consequential reduction of BMP4 gradient is hypothesized to cause expansion of WNT compartment (crypt hyperplasia). Similar mechanisms were hypothesized to cause crypt hyperplasia in acute Celiac Disease (A-CD). (B) In A-CD we observed a reduction of Hh signaling, that was not followed by a reduction of BMP4 levels. Nevertheless, we observed increase of WNT responding compartment. We hypothesized that IHH reduction is not a condition sufficient to reduce BMP4 protein levels in A-CD to drive crypt hyperplasia. However other molecular mechanisms can contribute to expansion of immature WNT compartment. Increased number of stromal cells known to be part of the Intestinal Stem Cell niche and increase levels of BMPs antagonists like GREM1 and GREM2, might play a relevant role in in A-CD crypt hyperplasia. [Adapted from Vanuytsel T et al., BBA, 2013].
(PDF)

S2 Fig *GREM2* Gene expression in HC and A-CD whole biopsies. Total RNA from HC (N = 10) and A-CD (N = 10) biopsies were evaluated by qRT-PCR. *GREM2* gene was found significantly upregulated in A-CD biopsies. (*) *P <* .*05*.
(PDF)

S3 Fig *In situ* hybridization of *LGR5* transcripts in HC and A-CD intestinal crypts. *LGR5* positive cells can be distinguished in high and low based on staining intensity. *LGR5* ^*high*^ labels the CBCs (Crypt Base Columnar Cells) (black arrow), whereas *LGR5* ^*low*^ identify immature proliferating (TA) progenitor cells. A significant increase of *LGR5* ^*low*^ positive cells was found in A-CD crypts (*C*, *D*) compared to healthy ones (*A*, *B*), no difference was found in the number of *LGR5* ^*high*^ cells. *Scale bar* = 50μm.
(PDF)

S4 Fig Volcano plot representing 80 stem cell related genes expression profile in celiac crypts. Samples from healthy (N **=** 5) and CD (N **=** 5) crypts were obtained by laser capture microscopy. Gene expression with a 2.5 fold of downregulation is represented with green dots and 2.5 upregulation is represented with red dot. Dots above the blue line represents genes expressed significantly (***P <* .*05***) different compared to control.
(PDF)
