# Supplementary figures and images for: Celiac Disease Histopathology Recapitulates Hedgehog Downregulation, Consistent with Wound Healing Processes Activation
Source: PLoS One. 2015 Dec 9;10(12):e0144634. doi: 10.1371/journal.pone.0144634 (PMC4674131; doi:10.1371/journal.pone.0144634)

S1 Fig.

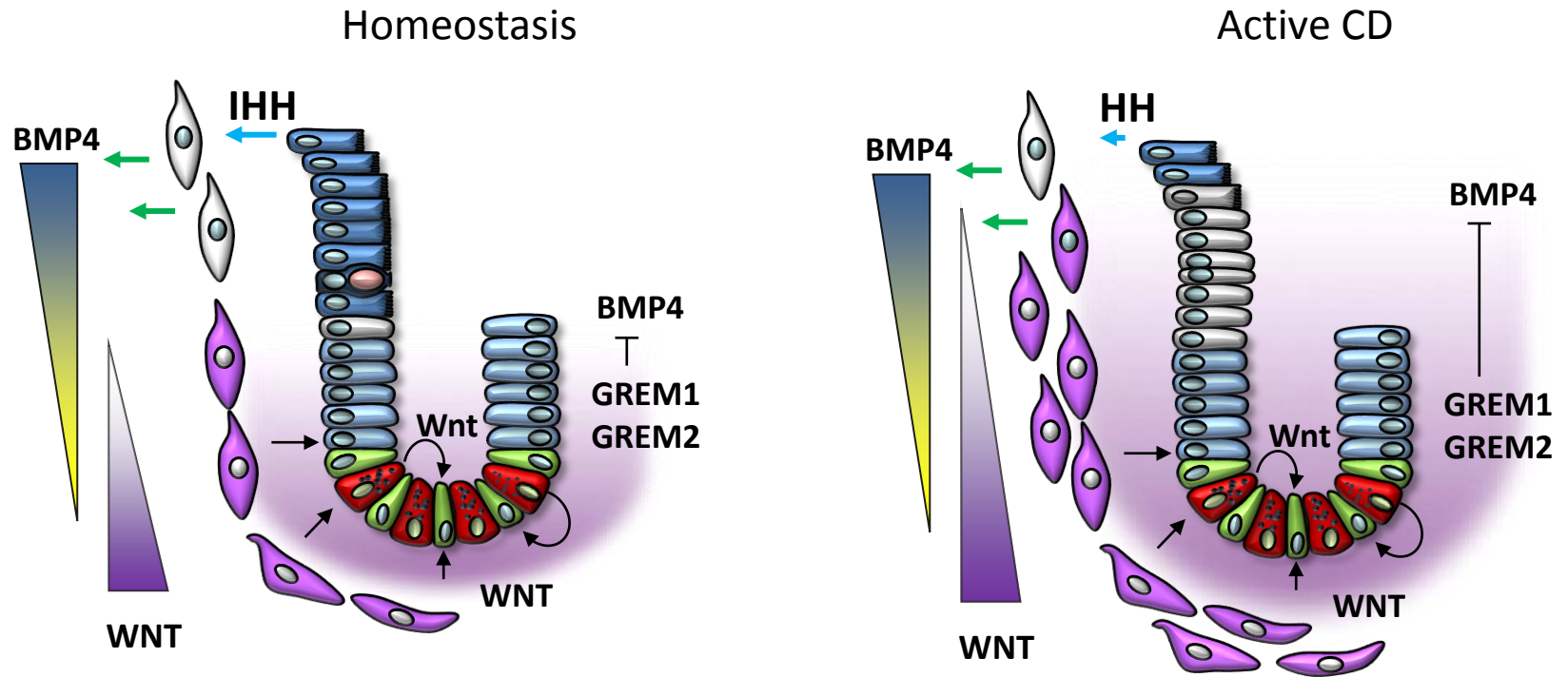

● Mature epithelial cells ● Intestinal stem cells ● Paneth's cells ● ISEMF

Supplement: S1 Fig — (A) At the homeostasis IHH is released by the mature epithelium and received by stromal cells that under IHH stimulus secrete BMP4. It is hypothesized that BMP4 gradient originating from stromal cells along the crypt villus axis opposes to WNT to restrain intestinal stem cell proliferation. WNT is released by Paneth cells and specialized ISEMFs (purple) that secrete also BMPs antagonists GREM1 and GREM2 providing a BMPs free stem cell niche. In Hh knockdown mouse a dampening of Hh signaling and consequential reduction of BMP4 gradient is hypothesized to cause expansion of WNT compartment (crypt hyperplasia). Similar mechanisms were hypothesized to cause crypt hyperplasia in acute Celiac Disease (A-CD). (B) In A-CD we observed a reduction of Hh signaling, that was not followed by a reduction of BMP4 levels. Nevertheless, we observed increase of WNT responding compartment. We hypothesized that IHH reduction is not a condition sufficient to reduce BMP4 protein levels in A-CD to drive crypt hyperplasia. However other molecular mechanisms can contribute to expansion of immature WNT compartment. Increased number of stromal cells known to be part of the Intestinal Stem Cell niche and increase levels of BMPs antagonists like GREM1 and GREM2, might play a relevant role in in A-CD crypt hyperplasia. [Adapted from Vanuytsel T et al., BBA, 2013]. (PDF) [file pone.0144634.s001.pdf]

S2 Fig.

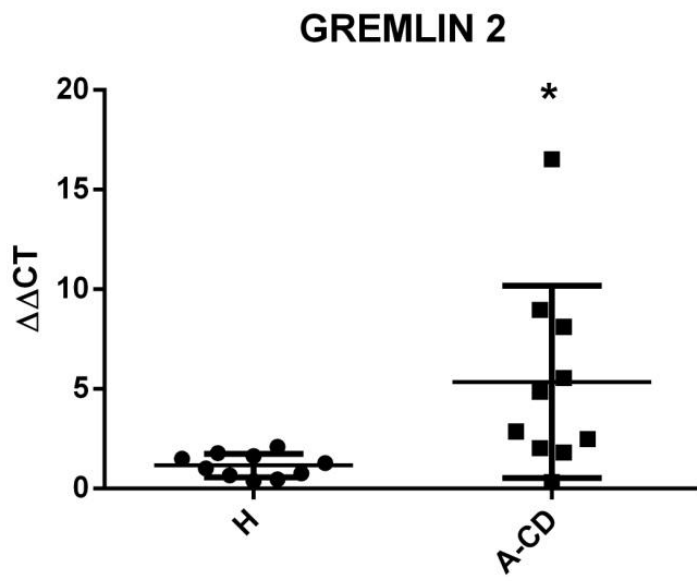

Supplement: S2 Fig — Total RNA from HC (N = 10) and A-CD (N = 10) biopsies were evaluated by qRT-PCR. GREM2 gene was found significantly upregulated in A-CD biopsies. (*) P < .05. (PDF) [file pone.0144634.s002.pdf]

S3 Fig.

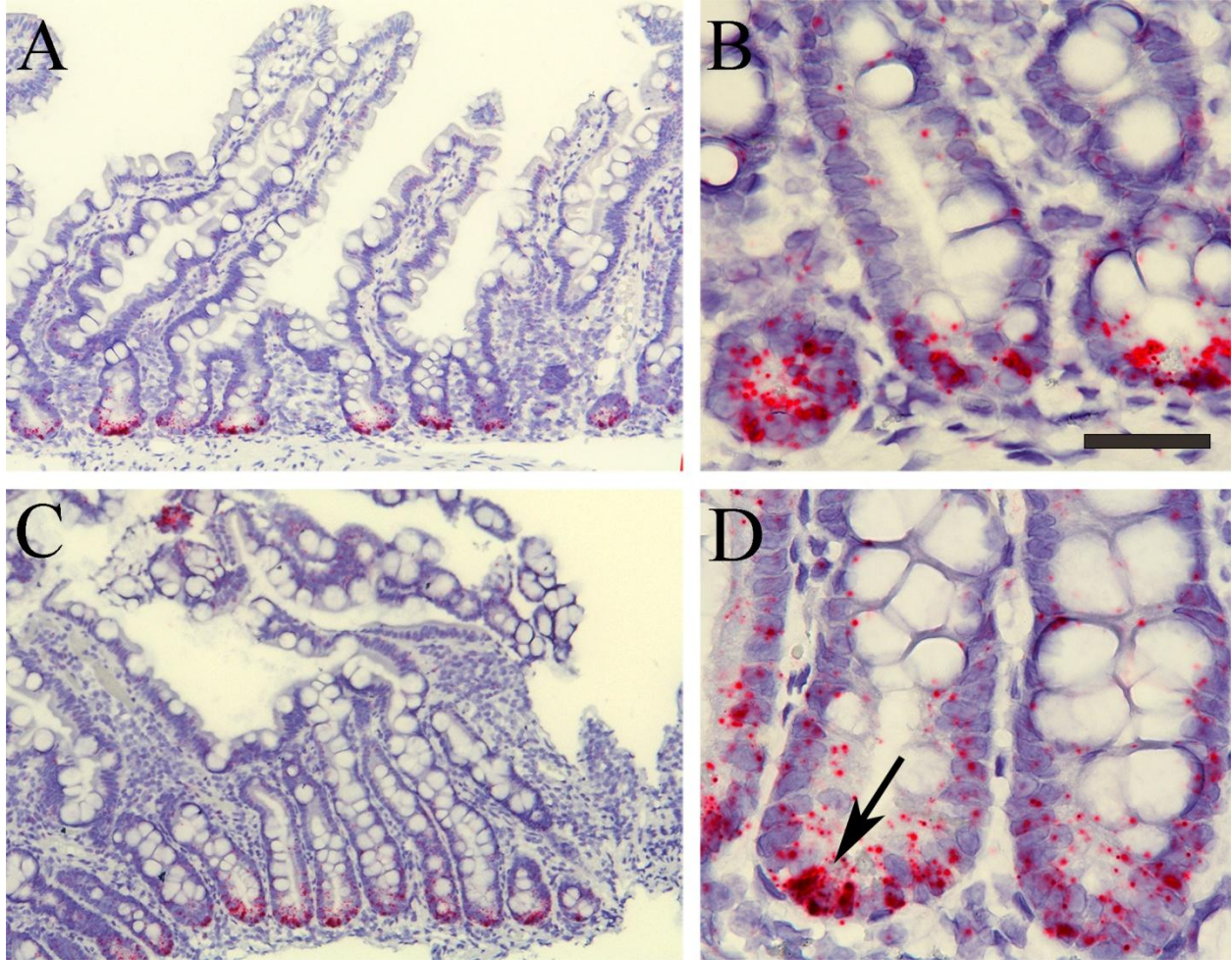

Supplement: S3 Fig — LGR5 positive cells can be distinguished in high and low based on staining intensity. LGR5 high labels the CBCs (Crypt Base Columnar Cells) (black arrow), whereas LGR5 low identify immature proliferating (TA) progenitor cells. A significant increase of LGR5 low positive cells was found in A-CD crypts (C, D) compared to healthy ones (A, B), no difference was found in the number of LGR5 high cells. Scale bar = 50μm. (PDF) [file pone.0144634.s003.pdf]

**S4 Fig.**

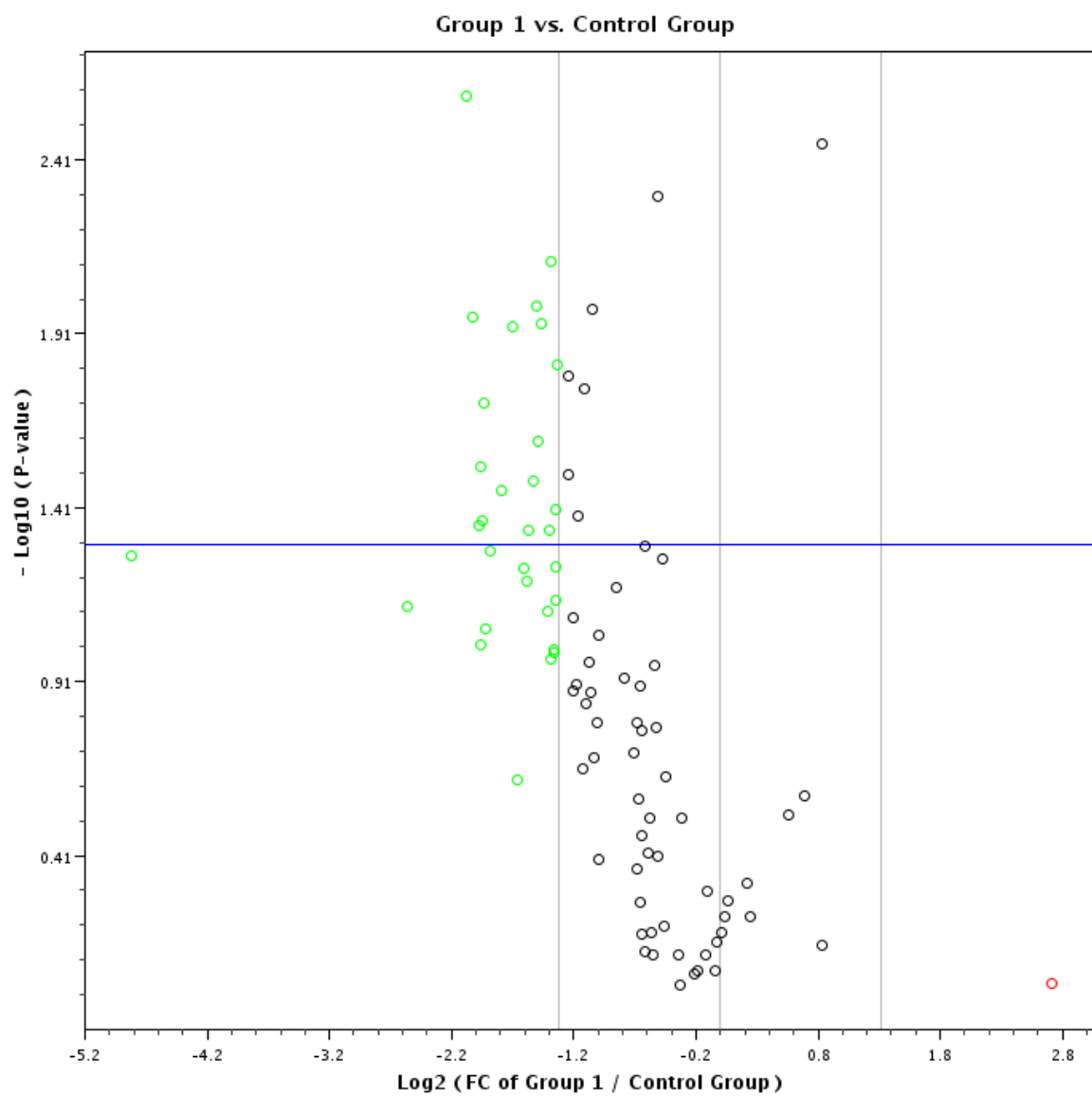

Supplement: S4 Fig — Samples from healthy (N = 5) and CD (N = 5) crypts were obtained by laser capture microscopy. Gene expression with a 2.5 fold of downregulation is represented with green dots and 2.5 upregulation is represented with red dot. Dots above the blue line represents genes expressed significantly (P < .05) different compared to control. (PDF) [file pone.0144634.s004.pdf]
